# Identification of PD-L1 Expression in Resectable NSCLC using Interpretable Machine Learning Model Based on Spectral CT

**DOI:** 10.2174/0115734056404160250925115913

**Published:** 2025-10-09

**Authors:** Henan Lou, Shiyu Cui, Yinying Dong, Shunli Liu, Shaoke Li, Hongzheng Song, Xiaodan Zhao

**Affiliations:** 1 Department of Radiology, The Affiliated Hospital of Qingdao University, Qingdao 266003, China; 2 Department of Radiation Oncology, The Affiliated Hospital of Qingdao University, Qingdao 266003, China; 3 Department of Radiology, Qingdao Municipal Hospital, Qingdao 266071, China

**Keywords:** Non-small cell lung cancer, X-ray computed Tomography, Programmed death ligand-1, Machine learning, Immune checkpoint inhibitors, SHAP

## Abstract

**Introduction:**

This study aimed to explore the value of a machine learning model based on spectral computed tomography (CT) for predicting the programmed death ligand-1 (PD-L1) expression in resectable non-small cell lung cancer (NSCLC).

**Methods:**

In this retrospective study, 131 instances of NSCLC who underwent preoperative spectral CT scanning were enrolled and divided into a training cohort (n = 92) and a test cohort (n = 39). Clinical-imaging features and quantitative parameters of spectral CT were analyzed. Variable selection was performed using univariate and multivariate logistic regression, as well as LASSO regression. We used eight machine learning algorithms to construct a PD-L1 expression predictive model. We utilized sensitivity, specificity, accuracy, calibration curve, the area under the curve (AUC), F1 score and decision curve analysis (DCA) to evaluate the predictive value of the model.

**Results:**

After variable selection, cavitation, ground-glass opacity, and CT40keV and CT70keV at venous phase were selected to develop eight machine learning models. In the test cohort, the extreme gradient boosting (XGBoost) model achieved the best diagnostic performance (AUC = 0.887, sensitivity = 0.696, specificity = 0.937, accuracy = 0.795 and F1 score = 0.800). The DCA indicated favorable clinical utility, and the calibration curve demonstrated the model’s high level of prediction accuracy.

**Discussion:**

Our study indicated that the machine learning model based on spectral CT could effectively evaluate the PD-L1 expression in resectable NSCLC.

**Conclusion:**

The XGBoost model, integrating spectral CT quantitative parameters and imaging features, demonstrated considerable potential in predicting PD-L1 expression.

## 1 INTRODUCTION

Lung cancer is a prevalent malignant neoplasm encountered in clinical practice, with non-small cell lung cancer (NSCLC) representing the most frequently observed type, comprising roughly 85% of all cases [1, 2]. Lung cancer persists as the principal cause of cancer-related mortality globally, despite the fact that low-dose chest CT scanning has become widely used and significantly improved the detection rate of early-stage lung cancer [3]. Surgical resection remains the preferred treatment for early-stage NSCLC; however, patients still face a substantial risk of postoperative recurrence and metastasis [4]. Recently, the advent of immunotherapy has introduced a new treatment paradigm. Immune checkpoint inhibitors (ICIs), particularly those targeting programmed death 1 (PD-1)/programmed death ligand 1 (PD-L1), have shown promising efficacy in managing operable early-stage NSCLC. Clinical studies have demonstrated that preoperative neoadjuvant immunotherapy can significantly reduce recurrence rates and improve survival rates of patients [5-7].

PD-L1 expression serves as a critical biomarker of ICIs in lung cancer [8, 9]. The selection of appropriate immunotherapy regimens largely depends on the PD-L1 expression level of the tumor. Currently, PD-L1 expression is predominantly assessed through immunohistochemical analysis of tissue obtained from needle biopsies. However, this invasive approach carries risks of complications, including bleeding and pneumothorax. Moreover, needle biopsies are limited by the potential for sampling bias due to the limited quantity of biopsy specimens and tumor heterogeneity, which may affect the accuracy of the results [10, 11]. Therefore, there is an urgent need for noninvasive and accurate methods to evaluate PD-L1 expression.

Spectral CT imaging, a novel scanning modality, demonstrates the morphological characteristics of tumors and allows for the quantitative analysis of tumors through parameters such as monochromatic CT values, slope of spectral curve (λHU), and iodine concentration (IC) [12, 13]. Recent studies have highlighted its potential in assessing Ki-67 expression and various gene mutations [14-16]. However, little is known about the utility of spectral CT in evaluating PD-L1 expression in NSCLC, and the relationship between spectral parameters and PD-L1 expression remains unclear.

This study aimed to build a model based on spectral CT using different machine learning algorithms to predict the PD-L1 expression in resectable NSCLC. Furthermore, we elucidated the interpretability of the model by the Shapley Additive exPlanations (SHAP) analysis.

## 2 MATERIALS AND METHODS

### 2.1 Patients

The ethics committee of the Affiliated Hospital of Qingdao University approved this retrospective study, and the requirement for patient-informed consent was waived. The study involved patients with NSCLC who accepted spectral CT exams from November 2020 to December 2022. Inclusion criteria included: (1) NSCLC confirmed by pathology after surgery and accepted PD-L1 immunohistochemical detection; (2) spectral CT scan performed within 30 days before surgery. Patients were excluded if they had received any anti-tumor therapy prior to the spectral CT scan or if the CT images were of poor quality. The patient selection process is shown in Fig. (**1**). The study enrolled 130 patients, with a total of 131 lesions.

### 2.2 CT Image Acquisition

All participants were scanned on a Revolution CT scanner (GE Healthcare, Chicago, USA). The scanning parameters were as follows: field of view, 500 mm; pitch, 0.992; tube current, automatic modulation; tube voltage, 80 kVp and 140 kVp instantaneous switching (0.5 ms); layer gap, 5 mm; layer thickness, 5 mm; reconstruction thickness, 1.25 mm. The contrast agent (Omnipaque, 350 mg I/mL, GE Healthcare) was injected intravenously at a rate of 3.0 mL/s at a dose of 70-80 mL. Following the injection of the contrast agent, the arterial phase (AP) was acquired at 25 seconds, and the venous phase (VP) was initiated at 60 seconds.

### 2.3 Image Analysis

The images were exported to a post-processing workstation (AW4.6, GE Healthcare), and analyzed using the GSI Viewer software. For each lesion, the largest axial slice of the tumor, together with the adjacent slices above and below, was selected for region of interest (ROI) delineation, carefully avoiding calcification and necrosis, large vessels and bronchi. The average value was taken as the final measurement value. An ROI was placed in the center of the aorta at the same level. The CT values on 100keV, 70keV and 40keV monochromatic images (CT100keV, CT70keV and CT40keV) and IC of each ROI at AP and VP were obtained. Normalized iodine concentration (NIC) and λHU were calculated as follows: NIC = IC_lesion_/IC_aorta_, λHU = (CT40keV - CT100keV)/(100–40).

### 2.4 Imaging Feature Assessment

Two radiologists independently reviewed the images without prior knowledge of the pathological findings. Any discrepancies were resolved through discussion. Imaging features included tumor diameter, location, spiculation, cavitation, lobulation, pleural indentation, and ground-glass opacity (GGO).

### 2.5 PD-L1 Expression Assay

Pathological specimens were preserved in 10% formalin, embedded in paraffin, and subjected to immunohistochemical staining using the 22C3 antibody (Dako company). The PD-L1 expression level was assessed utilizing the tumor proportion score (TPS). TPS < 1% was defined as negative and TPS ≥ 1% as positive.

### 2.6 Development and Validation of a Model

To identify factors for inclusion in the model, univariate logistic regression analysis was first performed to assess associations with PD-L1 expression. Subsequently, significant variables (*p* < 0.05) were incorporated into the multivariate logistic regression analysis using backward stepwise regression. To further mitigate potential confounding and prevent overfitting, the least absolute shrinkage and selection operator (LASSO) regression analysis was also applied for variable selection. Eight machine learning algorithms, including random forest, support vector machine (SVM), light gradient boosting machine (LightGBM), multilayer perceptron (MLP), logistic regression (LR), adaptive boosting (AdaBoost), extreme gradient boosting (XGBoost) and NaiveBayes, were applied to build the machine learning models. All algorithms were trained utilizing a 10-fold cross-validation strategy. The area under the receiver operating characteristic curve (AUC), sensitivity, specificity, accuracy and F1 score were utilized to gauge the predictive capability of the model. Calibration curve and decision curve analysis (DCA) were utilized to determine the goodness of fit and clinical efficacy of each model. Moreover, we employed the SHAP approach to explain the optimal prediction model, thereby illustrating the contribution and predictive significance of the chosen variables within the model.

### 2.7 Statistical Analysis

SPSS 26.0 software and Python software (3.9.7, www.python.org) were used to analyze the data. Statistical analyses included the Fisher's exact test or chi-squared test employed for categorical data and the two-sample t test or Mann-Whitney U test employed for continuous data. Values with *p* < 0.05 were considered statistically significant.

## 3 RESULTS

### 3.1 Data Cohort Information

The study cohort contained 130 NSCLC patients with 131 identifiable lesions (PD-L1 negative/PD-L1 positive, 68/63). Patients included 63 women and 67 men aged 45 to 79 years (mean age ± standard deviation, 60.66 ± 6.67 years). According to a 7:3 ratio, the training group consisted of 92 randomly selected patients, whereas the test cohort consisted of 38 individuals (39 lesions). All characteristics and parameters were evenly distributed between the two cohorts (Table **1**).

### 3.2 Screening of Variables

The outcomes of the univariate logistic regression analysis revealed a significant correlation between PD-L1 expression and 12 variables. These variables were further analyzed using multivariate logistic regression analysis, which identified four key predictors: cavitation, GGO, CT40keV and CT70keV at VP (Table **2**). Meanwhile, LASSO regression analysis was applied to 19 candidate variables and selected 12 relevant variables, including tumor diameter, location, pleural indentation, cavitation, GGO, VP-IC, and CT40keV, CT70keV and CT100keV at AP and VP. This finding indicates no significant multicollinearity among the selected variables.

### 3.3 Model Construction and Evaluation

**Fig. (2):**
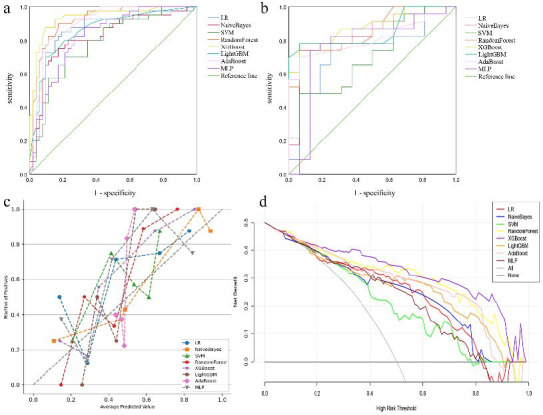
Model performance analysis. ROC curves of different machine learning models for identifying PD-L1 expression in the training (**a**) and test cohorts (**b**). (**c**) Calibration curves for each model in the test cohort. (**d**) Decision curve analysis of each model. SVM, support vector machine; LightGBM, light gradient boosting machine; MLP, multilayer perceptron; LR, logistic regression; AdaBoost, adaptive boosting; XGBoost, extreme gradient boosting.

Utilizing the four key variables identified by multivariate regression, we constructed the machine learning models with eight distinct algorithms. Our findings indicated the XGBoost model achieved the highest AUC values in the training and test sets (Fig **2a**,**b**). The performance metrics of the XGBoost model in the training cohort were as follows: an AUC value of 0.937, a sensitivity of 0.825, a specificity of 0.923, an accuracy of 0.880 and an F1 score of 0.857. Further examination of the data in the test cohort indicated that the XGBoost model exhibited an AUC value of 0.887, a sensitivity of 0.696, a specificity of 0.937, an accuracy of 0.795 and an F1 score of 0.800 (Table **3**). These results highlight its strong predictive capability. The calibration curve revealed that the NaiveBayes and XGBoost models had high predictive accuracy (Fig. **2c**). DCA indicated that the XGBoost model possessed the greatest clinical utility (Fig. **2d**). Having conducted a comprehensive analysis of these results, we believe that the XGBoost model stands out as the optimal prediction model.

### 3.4 Model Interpretation

**Fig. (3):**
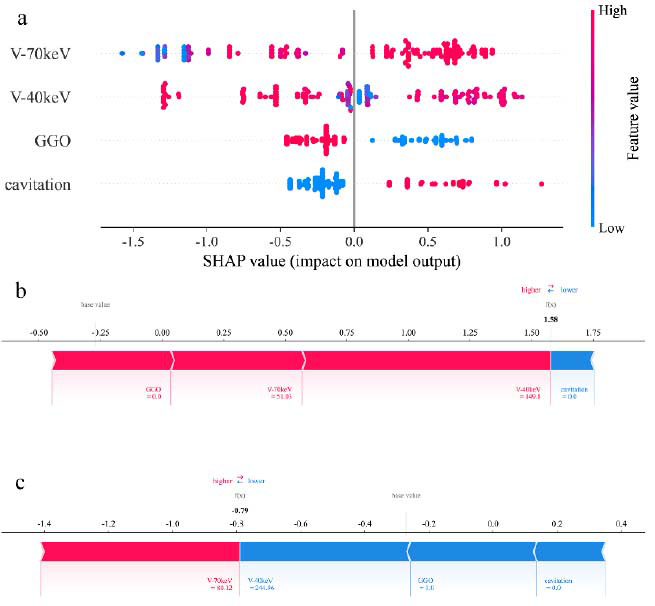
SHAP interpretation of the model. (**a**) Characteristic attributions in SHAP. Each line corresponds to a variable, with the X-axis indicating the SHAP value. Blue dots denote lower eigenvalues, while red dots signify higher eigenvalues. Individual characteristics of a patient with PD-L1-positive (**b**) and PD-L1-negative NSCLC (**c**). SHAP values reflect the predictive features of the sample and the contribution of each feature to the prediction. The base value represents the model’s prediction in a scenario where no features are provided, while the figures in bold denote the final predicted score. The presence of red bars signifies features that positively impact the prediction, whereas blue bars denote those that have a negative effect. The extent of a feature’s influence is visualized through the length of the bars, with longer bars suggesting a more significant contribution to the prediction.

To elucidate the selected variables visually, we employed SHAP to clarify the importance of variables in differentiating PD-L1 expression within the XGBoost model. Fig. (**3a**) delineates the importance ranking of four pivotal variables in the model, where the influence of each variable is visualized through different colored dots-those in red signify high-risk attributes, while blue dots denote low-risk attributes. Two quantitative parameters (VP-CT40keV and VP-CT70keV) exhibited a strong correlation with PD-L1 expression, underscoring their diagnostic significance. Moreover, two representative cases are presented to illustrate the model’s interpretability: one involving a patient with PD-L1-positive NSCLC who had a high SHAP prediction score (1.58) (Fig. **3b**), and another case of a patient with PD-L1-negative NSCLC who exhibited a low SHAP score (-0.79) (Fig. **3c**). The corresponding spectral CT images for these cases are shown in Figs. (**4a**-**j** and **5a-j)**, respectively.

## 4 DISCUSSION

In our study, eight machine learning algorithms were utilized to develop predictive models integrating spectral CT quantitative parameters and imaging features for PD-L1 expression identification. The XGBoost model displayed good diagnostic capability (AUC = 0.887, in the test cohort) and clinical application value, offering a promising tool to support clinicians in selecting therapeutic strategies.

Immunotherapy has become a crucial therapeutic approach, demonstrating significant clinical efficacy in advanced NSCLC as well as promising outcomes in early-stage cancer management. In particular, PD-L1 ICIs have been shown to confer significant survival benefits in NSCLC patients [7, 17]. The implementation of this therapy highlights the clinical need for accurate PD-L1 expression evaluation to identify which patients will benefit from treatment. In our study, the predictive model, integrating quantitative parameters with imaging features, demonstrated robust discriminative capacity in distinguishing PD-L1-positive from PD-L1-negative tumors, potentially aiding in ICIs selection.

CT value serves as a quantitative indicator of lesion density, providing essential information for assessing both the degree of contrast enhancement and the relative vascular perfusion characteristics of tumors. In our study, univariate analysis revealed that CT100keV, CT70keV, and CT40keV at both AP and VP displayed a significant association with PD-L1 expression, consistent with Chen *et al*. [18] and Wang *et al*. [19]. Biologically, PD-L1 expression can trigger the PD-1/PD-L1 signaling pathway, which in turn suppresses T cell-mediated immune responses, enabling tumor cells to escape immune detection. This mechanism contributes to tumor progression and necessitates increased blood supply and nutrition to support the growing tumor mass [20, 21]. Consequently, these findings reflect the more aggressive nature of PD-L1-positive tumors and the more abundant blood supply that can be observed. Notably, our analysis identified VP-CT40keV and VP-CT70keV as independent predictors of PD-L1 expression compared to AP parameters. Spectral parameters can reflect tumor microvascular density. The complex, tortuous architecture of tumor microvasculature results in slower contrast agent circulation. During the arterial phase following contrast injection, the contrast agent may not fully perfuse the microvascular network. In contrast, the delayed venous phase imaging allows for more complete contrast filling of these microvessels [22]. These factors likely contribute to the observed differences between contrast phases.

This study demonstrated that cavitation and GGO are useful for assessing PD-L1 expression. PD-L1-positive tumors typically exhibit higher proliferative properties and when the tumors outgrow the available blood supply, this can result in the formation of cavitation [23]. Our results suggest that GGO loss was connected to positive PD-L1 expression, aligning with previous studies [23, 24], in which the presence of GGO was indicative of lower tumor aggressiveness [25]. These findings underscore the correlation between PD-L1 expression and the aggressive nature of tumors.

To more effectively assess PD-L1 expression, several studies have developed predictive models. Another study constructed a parameter-clinical nomogram that incorporated seven predictive factors and achieved AUC values of 0.824 and 0.825 in two separate validation groups, supporting the utility of quantitative parameters in predicting PD-L1 expression [19]. Liu *et al*. [26] constructed a model using deep-learning signature and clinicopathological factors for predicting PD-L1 expression, which achieved an AUC value of 0.804 in the validation set. In this study, we developed spectral CT-based predictive models for assessing PD-L1 expression in NSCLC using eight machine learning algorithms. The optimal model demonstrated commendable predictive capability (AUC = 0.887), which was superior to their models. XGBoost represents an efficient machine learning algorithm based on a gradient boosting framework, which incorporates a regularization technique to effectively avoid overfitting while showing superior performance in handling complex classification tasks [27]. Additionally, we employed the SHAP approach to interpret the predictive significance of each variable within the model. The results revealed that two quantitative parameters emerged as the predominant factors within the model, highlighting their pivotal role in the prediction process. Chen *et al.* [28] demonstrated a moderate positive correlation between CT70keV and CT40keV and Ki-67 index, indicating that these spectral parameters can indirectly reflect the proliferative activity of the tumor. PD-L1-positive tumors exhibit strong invasiveness and active cell proliferation; thus, employing spectral parameters to evaluate PD-L1 expression holds potential application value.

## 5 LIMITATION

This study presents a promising evaluation tool for clinical practice, but several limitations remain. First, as a single-center, retrospective analysis, it carries the risk of selection bias and may limit the generalization of our results; and multicenter studies should be considered in the future to verify the robustness of the model. Second, the sample size is small, and a larger dataset is required to validate our findings. Finally, the use of a single scanning device in this study may restrict the applicability of our results in clinical settings.

## CONCLUSION

The XGBoost model developed in this study demonstrated superior diagnostic performance in evaluating PD-L1 expression in NSCLC patients, and could offer valuable support for clinicians in developing individualized treatment strategies.

## Figures and Tables

**Fig. (1):**
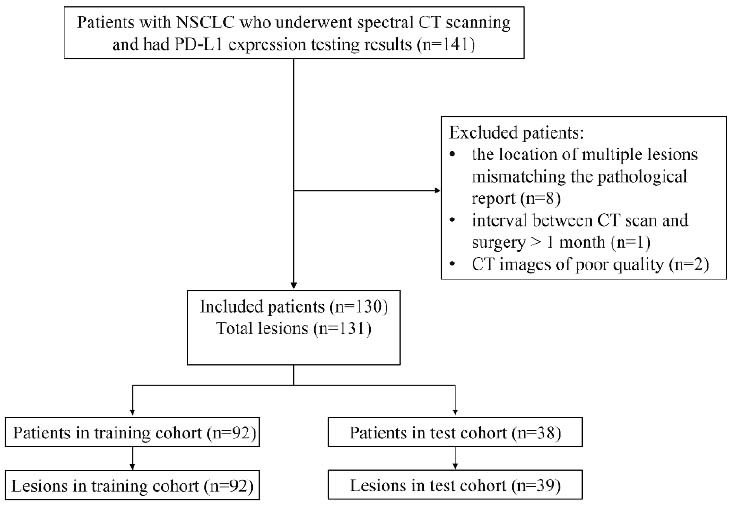
Flowchart of patient selection.

**Fig. (4):**
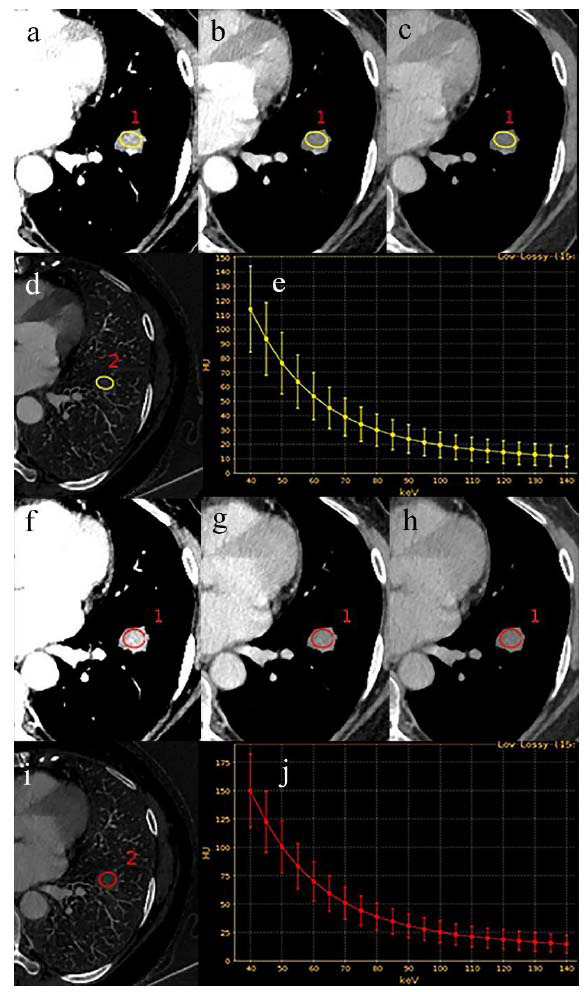
Spectral CT imaging of a 53-year-old woman with PD-L1-positive lung adenocarcinoma. (**a-c**) The arterial phase CT 40keV, CT 70keV and CT 100keV values were 113.96 HU, 39.00 HU and 19.57 HU, respectively. (**d**) Arterial phase iodine base map, IC = 13.37 × 100 μg/cm^3^ (**e**) Arterial phase spectral curve, λHU = 1.57. (**f-h**) The venous phase CT 40keV, CT 70keV and CT 100keV values were 149.80 HU, 51.03 HU and 25.41 HU, respectively. **i** Venous phase iodine base map, IC = 17.68 × 100 μg/cm^3^. (**j**) Venous phase spectral curve, λHU = 2.07.

**Fig. (5):**
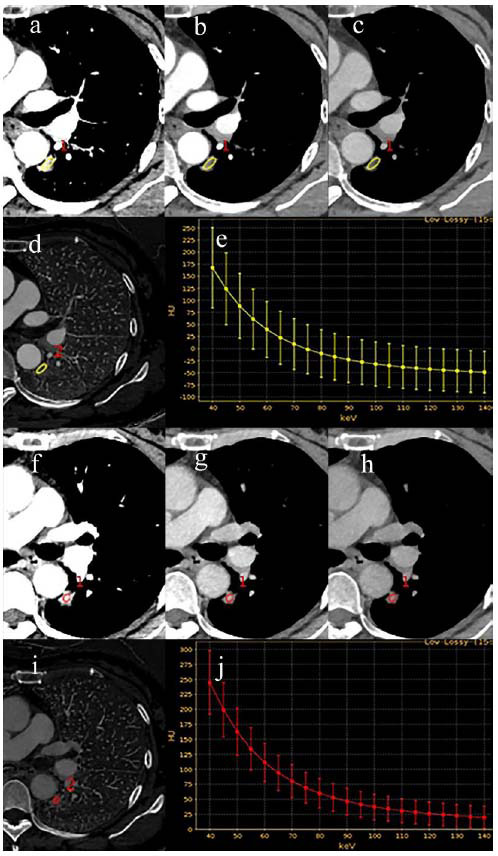
Spectral CT imaging of a 53-year-old woman with PD-L1-negative lung adenocarcinoma. (**a**-**c**) The arterial phase CT 40keV, CT 70keV and CT 100keV values were 167.44 HU, 9.19 HU and -32.00 HU, respectively. (**d)** Arterial phase iodine base map, IC = 28.01 × 100 μg/cm^3^. **e** Arterial phase spectral curve, λHU = 3.32. **(f-h)** The venous phase CT 40keV, CT 70keV and CT 100keV values were 244.96 HU, 80.12 HU and 37.35 HU, respectively. **(i)** Venous phase iodine base map, IC = 29.20 × 100 μg/cm^3^. (**j)** Venous phase spectral curve, λHU = 3.46.

**Table 1:** Distribution of quantitative parameters and clinical-imaging features in the two cohorts.

**Variables**	**Training Cohort**	**Test Cohort**	***p*-value**
Patient features			0.873
Sex, n (%)			
Men	47 (51.1)	20 (52.6)	
Women	45 (48.9)	18 (47.4)	
Age (y)	60.38 ± 6.66	61.34 ± 6.73	0.457
Tumor features			0.812
Pathology type			
Adenocarcinoma	84 (91.3)	35 (89.7)	
Squamous cell carcinoma	7 (7.6)	4 (10.3)	
Others	1 (1.1)	0 (0.0)	
PD-L1 expression, n (%)			0.105
Positive	40 (43.5)	23 (59.0)	
Negative	52 (56.5)	16 (41.0)	
Tumor diameter (cm)	2.33 ± 1.18	2.31 ± 0.97	0.951
Location, n (%)			0.364
Right upper lobe	43 (46.7)	11 (28.2)	
Right lower lobe	13 (14.1)	7 (17.9)	
Right middle lobe	5 (5.4)	3 (7.7)	
Left upper lobe	20 (21.7)	12 (30.8)	
Left lower lobe	11 (12.0)	6 (15.4)	
Spiculation, n (%)			0.839
Absence	36 (39.1)	16 (41.0)	
Presence	56 (60.9)	23 (59.0)	
Lobulation, n (%)			0.204
Absence	11 (12.0)	8 (20.5)	
Presence	81 (88.0)	31 (79.5)	
Cavitation, n (%)			0.672
Absence	70 (76.1)	31 (79.5)	
Presence	22 (23.9)	8 (20.5)	
Pleural indentation, n (%)			0.073
Absence	22 (23.9)	-10.3	
Presence	70 (76.1)	35 (89.7)	
GGO, n (%)			0.908
Absence	34 (37.0)	14 (35.9)	
Presence	58 (63.0)	25 (64.1)	
AP parameters			
CT40keV	129.43 (170.25)	140.34 (109.39)	0.341
CT70keV	44.34 (181.45)	48.93 (53.38)	0.3
CT100keV	14.08 (172.91)	13.77 (62.21)	0.336
λHU	2.45 ± 1.11	2.53 ± 1.18	0.728
IC	20.71 ± 9.40	21.34 ± 9.95	0.729
NIC	0.20 (0.15)	0.19 (0.15)	0.88
VP parameters			
CT40keV	168.37 (200.70)	171.61 (94.83)	0.553
CT70keV	59.48 (177.14)	55.51 (62.84)	0.556
CT100keV	25.45 (174.47)	23.64 (52.53)	0.51
λHU	2.50 ± 1.02	2.51 ± 0.99	0.983
IC	21.12 ± 8.62	21.17 ± 8.31	0.972
NIC	0.51 ± 0.19	0.51 ± 0.22	0.996

**Note:** Data were expressed as median (interquartile range) or mean ± standard deviation. PD-L1, programmed death ligand-1; GGO, ground-glass opacity; AP, arterial phase; VP, venous phase; IC, iodine concentration; λHU, slope of the spectral curve; NIC, normalized iodine concentration.

**Table 2:** Feature screening in univariate and multivariate logistic regression analyses.

**Variable**	**Univariate Analysis**			**Multivariate Analysis**		
	**OR**	**95%CI**	***p*-value**	**OR**	**95%CI**	***p*-value**
Tumor diameter	2.006	1.235-3.258	0.005	-	-	-
Location	1.171	0.893-1.535	0.255	-	-	-
Spiculation	2.441	1.011-5.896	0.047	-	-	-
Lobulation	1.400	0.380-5.159	0.613	-	-	-
Cavitation	3.857	1.388-10.715	0.010	6.009	1.583-22.817	0.008
Pleural indentation	1.148	0.434-3.035	0.781	-	-	-
GGO	0.198	0.079-0.495	0.001	0.317	0.106-0.946	0.039
AP parameters CT40keV	1.002	1.000-1.005	0.039	-	-	-
CT70keV	1.004	1.001-1.007	0.009	-	-	-
CT100keV	1.005	1.001-1.008	0.006	-	-	-
λHU	0.638	0.423-0.964	0.033	-	-	-
IC	0.949	0.904-0.996	0.034	-	-	-
NIC	0.020	0.000-1.461	0.074	-	-	-
VP parameters CT40keV	1.002	1.000-1.004	0.042	0.981	0.968-0.994	0.004
CT70keV	1.004	1.001-1.007	0.012	1.026	1.010-1.042	0.001
CT100keV	1.004	1.001-1.007	0.008	-	-	-
λHU	0.709	0.464-1.085	0.113	-	-	-
IC	0.960	0.913-1.010	0.113	-	-	-
NIC	0.462	0.053-4.048	0.486	-	-	-

**Abbreviations:** CI, confidence interval; OR, odds ratio; GGO, ground-glass opacity; AP, arterial phase; VP, venous phase; IC, iodine concentration; λHU, slope of the spectral curve; NIC, normalized iodine concentration.

**Table 3:** Performance of different machine learning models in the training and test cohorts.

**Cohort**	**Model**	**AUC (95%CI)**	**Sensitivity**	**Specificity**	**Accuracy**	**F1 score**
Training cohort	LR	0.853 (0.769-0.938)	0.825	0.808	0.815	0.795
	RF	0.918 (0.861-0.975)	0.875	0.827	0.848	0.833
	LightGBM	0.858 (0.779-0.937)	0.675	0.904	0.804	0.750
	SVM	0.781 (0.686-0.876)	0.675	0.788	0.739	0.692
	XGBoost	0.937 (0.886-0.989)	0.825	0.923	0.880	0.857
	NaiveBayes	0.819 (0.728-0.911)	0.725	0.827	0.783	0.744
	MLP	0.826 (0.743-0.910)	0.800	0.731	0.761	0.744
	AdaBoost	0.905 (0.844-0.966)	0.775	0.885	0.837	0.805
Test cohort	LR	0.742 (0.577-0.906)	0.739	0.750	0.744	0.773
	RF	0.855 (0.738-0.971)	0.696	0.937	0.795	0.800
	LightGBM	0.856 (0.734-0.978)	0.739	0.937	0.821	0.829
	SVM	0.704 (0.537-0.870)	0.435	0.937	0.641	0.588
	XGBoost	0.887 (0.786-0.989)	0.696	0.937	0.795	0.800
	NaiveBayes	0.829 (0.690-0.968)	0.696	0.937	0.795	0.800
	MLP	0.772 (0.598-0.946)	0.696	0.875	0.769	0.780
	AdaBoost	0.807 (0.668-0.946)	0.609	0.937	0.744	0.737

**Abbreviations:** CI, confidence interval; AUC, area under the curve; AP, arterial phase; VP, venous phase. LR, logistic regression; RF, randomforest; LightGBM, light gradient boosting machine; SVM, support vector machine; XGBoost, extreme gradient boosting; MLP, multilayer perceptron; AdaBoost, adaptive boosting.

## Data Availability

The data analyzed in this study are available from the corresponding authors [H.S] and [X.Z] upon reasonable request.

## LIST OF ABBREVIATIONS

CT: Computed tomography
PD-L1: Programmed death ligand-1
AUC: Area under the curve
NSCLC: Non-small cell lung cancer
ICIS: Immune checkpoint inhibitors
IC: Iodine concentration
SHAP: Shapley Additive exPlanations
AP: Arterial phase
ROI: Region of interest
GGO: Ground-glass opacity
TPS: Tumor proportion score
LASSO: Least absolute shrinkage and selection operator
SVM: Support vector machine
MLP: Multilayer perceptron
DCA: Decision curve analysis

## STANDARDS OF REPORTING

STROBE guidelines were followed.
